# Interactions of the *Streptococcus pneumoniae* Toxin-Antitoxin RelBE Proteins with Their Target DNA

**DOI:** 10.3390/microorganisms9040851

**Published:** 2021-04-15

**Authors:** Inmaculada Moreno-Córdoba, Wai-Ting Chan, Concha Nieto, Manuel Espinosa

**Affiliations:** Centro de Investigaciones Biológicas Margarita Salas, CSIC, Ramiro de Maeztu, 9, E28040 Madrid, Spain; chanyting@hotmail.com (W.-T.C.); cnieto@cib.csic.es (C.N.)

**Keywords:** toxin-antitoxin systems, pneumococcal RelBE, protein-DNA interactions, DNA footprints, transcriptional fusions

## Abstract

Type II bacterial toxin-antitoxin (TA) systems are found in most bacteria, archaea, and mobile genetic elements. TAs are usually found as a bi-cistronic operon composed of an unstable antitoxin and a stable toxin that targets crucial cellular functions like DNA supercoiling, cell-wall synthesis or mRNA translation. The type II RelBE system encoded by the pathogen *Streptococcus pneumoniae* is highly conserved among different strains and participates in biofilm formation and response to oxidative stress. Here, we have analyzed the participation of the RelB antitoxin and the RelB:RelE protein complex in the self-regulation of the pneumococcal *relBE* operon. RelB acted as a weak repressor, whereas RelE performed the role of a co-repressor. By DNA footprinting experiments, we show that the proteins bind to a region that encompasses two palindromic sequences that are located around the −10 sequences of the single promoter that directs the synthesis of the *relBE* mRNA. High-resolution footprinting assays showed the distribution of bases whose deoxyriboses are protected by the bound proteins, demonstrating that RelB and RelB:RelE contacted the DNA backbone on one face of the DNA helix and that these interactions extended beyond the palindromic sequences. Our findings suggest that the binding of the RelBE proteins to its DNA target would lead to direct inhibition of the binding of the host RNA polymerase to the relBE promoter.

## 1. Introduction

Chromosomally-encoded Type II Toxin-Antitoxin bacterial systems (TAs) are constituted by a pair of genes organized as an operon in which the gene encoding the antitoxin usually precedes the gene encoding its cognate toxin [1,2,3]. Under steady-state growth conditions, the toxin is neutralized by the unstable antitoxin generating a tight and harmless protein–protein complex. However, when the cells encounter stressful situations, the antitoxins are degraded by host proteases (Lon or Clp) releasing the cognate toxin molecules that are stable and would exert their poisonous role [4,5,6,7,8]. Toxin activeness usually brings about a cell response that leads to a severe reduction in the metabolism, and as a consequence, a dormant state also known as persistence [9]. TAs are ubiquitous in the bacterial world, and they may be present in multiple copies in the bacterial chromosome, posing the question of why there is a need for such genetic redundancy [10]. Several roles have been reported for these genetic modules, such as (i) plasmid stability systems; (ii) genetic stabilization; (iii) defence against bacteriophage infection by causing abortive infection, and (iv) persistence, among others [11]. However, several of the roles ascribed to TAs have been recently revisited, and a conclusion that involvement of TAs in the regulation of bacterial lifestyle lack solid evidence has been proposed (see reviews in References [12,13,14,15]). Instead, a selfish qualification of these bacterial operons could lead to some of the adduced roles of TAs, permitting the chromosomal TAs to endure without providing any substantial advantage to their hosts [14]. Further, TAs encoded by bacterial pathogens are coming up as involved in their pathogenesis, since they participate in biofilm formation, oxidative stress, and persistence, which is a transient stasis situation that renders the bacterial cell immune to antibiotic killing [16]. Moreover, TAs are involved in the inhibition of virulence genes, either by antitoxin-mediated modulation of their synthesis or by toxin-mediated cleavage of virulence-encoding mRNAs [17,18,19]. Another role for TAs, which has recently been discovered, has shown that the plasmid-encoded TA PrpT/PrpA from *Pseudoalteromonas rubra* actively participates in the control of the plasmid copy number [20], adding a new level of complexity to these operons and constituting another example of cross-talks between separated plasmid modules [21].

One of the most abundant members among the TAs is represented by the RelBE pair family, as shown earlier [3]. They are widespread among bacteria and archaea, representing perhaps the most populated family of TAs [13,22]. The toxins (RelE) of this family is an effective ribonuclease that degrades essential RNAs, thus inhibiting bacterial cell growth; in particular, RelE toxins were shown to cleave mRNAs positioned at the ribosomal A-site [23,24]. Transcriptional regulation of the operon is exerted by their DNA binding abilities to an operator region. The two proteins behaved as co-repressors in the sense that a dimer of the RelB antitoxin would repress transcription of the operon; however, when toxin RelE is synthesized, it acts as an efficient co-repressor. At high RelE concentrations, the excess of toxin molecules could destabilize the RelB::RelE complex bound at the operator, a mechanism defined as ‘conditional cooperativity’ [25].

In the case of the Gram-positive pathogenic bacterium *Streptococcus pneumoniae* (the pneumococcus), up to ten different putative type II-TAs were detected by bioinformatics approaches [1]. This was somewhat surprising, given the economy of the pneumococcal genome [26]. However, experimental (‘wet’) studies showed that only four of them are functional at least in the laboratory conditions tested, namely PezAT [27,28], RelBE [29], YefMYoeB [30,31], and later on, PhDDoc [32]. These TAs were conserved in all or several of the pneumococcal genomes analyzed, although conservation was much higher in the case of the toxins than in the antitoxins [1,6], a feature that seems to be general among the bacterial genomes [13,15,33]. Out of the pneumococcal RelBE family, three operons were found in the chromosome of strain R6 [1,26]: two copies of *relBE* and one copy of *yefMyoeB* [34,35]. One of the *relBE* copies (*relBE1Spn*) was later shown to be non-functional, most likely due to changes in several amino acid residues that are critical for toxicity [32]. The pneumococcal *relBE* was the only TA pair found to be present in the chromosomes of all pneumococcal strains. However, the genomic organization of the region showed variations among the different strains, allowing us to define up to six different genomic distributions [1,36]. In four of them, the operon is placed just downstream of the *vicX* gene (metal-dependent hydrolase), followed by a more variable region that includes an enigmatic pneumococcal repeat, the 107-nt-long repeat unit of pneumococcus, the RUP unit [37], and genes *ldh* (lactate dehydrogenase) and *gyrA* (the A subunit of DNA gyrase). Other observed variations include: (i) type-II restriction/modification module; (ii) gene encoding a K-cation channel protein, and (iii) *IS1167* or *IS1380* insertion sequences [1]. Several strains exhibited a second copy of the operon, apparently as a result of the acquisition of foreign DNA, and in these strains, the genetic organization of the chromosome at these regions were different, since copies of putative *xre*-COG2856B intact or truncated genes were found [1]. These findings suggested to us that the pneumococcal *relBE* TA have an active and relevant role in the bacterium lifestyle and that horizontal transfer of copies of these genes could take place [1]. By employment of deletion and complementation analyses, we demonstrated that the RelBE, in conjunction with the YefMYoeB pair, participated in biofilm formation and oxidative stress response, suggesting that both TAs cooperate in the control of virulence genes (adhesion among others) in the pneumococcus [38]. An in-depth study of the pneumococcal transcriptome under infection conditions showed that maximal expression of the pneumococcal RelBE proteins was reached after 30 min post-infection [39] suggesting that this TA system, rather than playing a parasitic role, participate actively in the infective cycle of the pneumococcus, although liberation of the toxin as a consequence of bacterial stresses during infection was not addressed. Very interestingly, it has been recently shown that various stress-inducers bring about transcription of TA operons without a concomitant release of significant amounts of active toxin [40]. 

In the present work, we have determined the presence of the *relE* toxin gene in the chromosome of the best characterized pneumococcal strains so far [41] and found a distribution of RelE proteins that grouped within three main families. Further, we have tested the ability of the RelB:RelE proteins to repress transcription from their promoter by in vivo transcriptional fusions. Using purified RelB protein alone or the RelB:RelE complex, we have analyzed the ability of the RelB:RelE protein–protein complex to interact with their target DNA and to precisely define their binding site(s). To do so, we have used DNase I and high-resolution hydroxyl-radical (OH·) footprinting experiments. The target of the proteins has been precisely determined and shown to be located on two palindromic sequences that encompass the −10 region and extends past the initiation of transcription point.

## 2. Materials and Methods

### 2.1. Bacterial Strains, Transformation, and Growth Conditions

**Figure 1:**
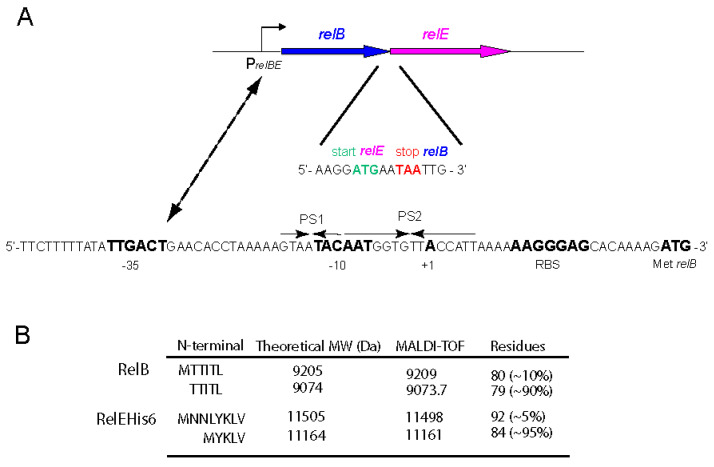
The *relBE* operon and the control region and features of the proteins it encodes. (**A**). Transcription of both genes is directed by a single promoter (P*_relBE_*) and the intervening sequence of both genes shows that the initiation codon of *relE* (green) is placed just before the stop codon of *relB* (red). The promoter P*_relBE_*, with the −35 and −10 boxes (boldface), the transcription start point (+1), the ribosome binding site (RBS) and the initiation codon of *relB* are indicated. The two palindromic sequences (PS) are indicated by arrows. (**B**). MALDI assays showing the N-terminal amino acid composition of the purified RelB and RelEHis_6_ proteins. In the case of antitoxin RelB, ~90% of the proteins showed processing of the initial Met residue, whereas only 5% of the purified toxin RelE exhibited the M-N-N residues extension at the N-terminal end of the protein.

**Figure 2:**
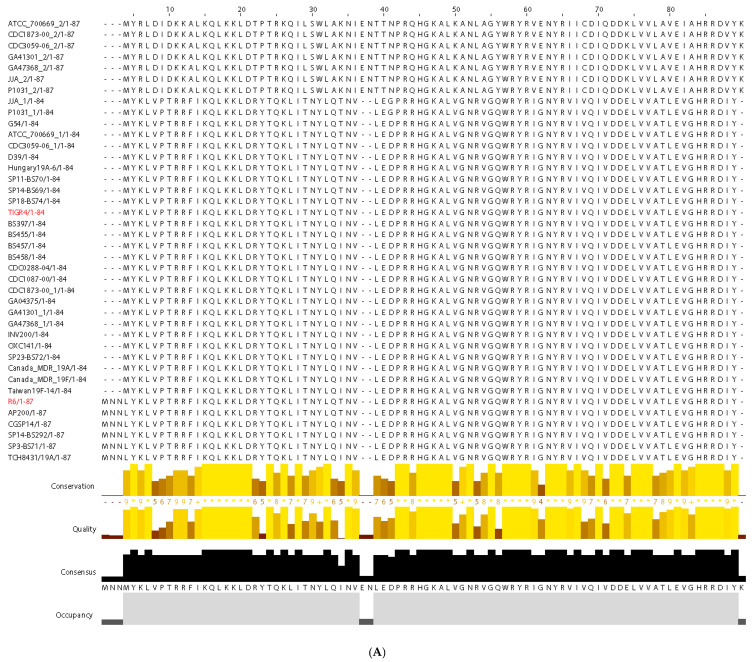

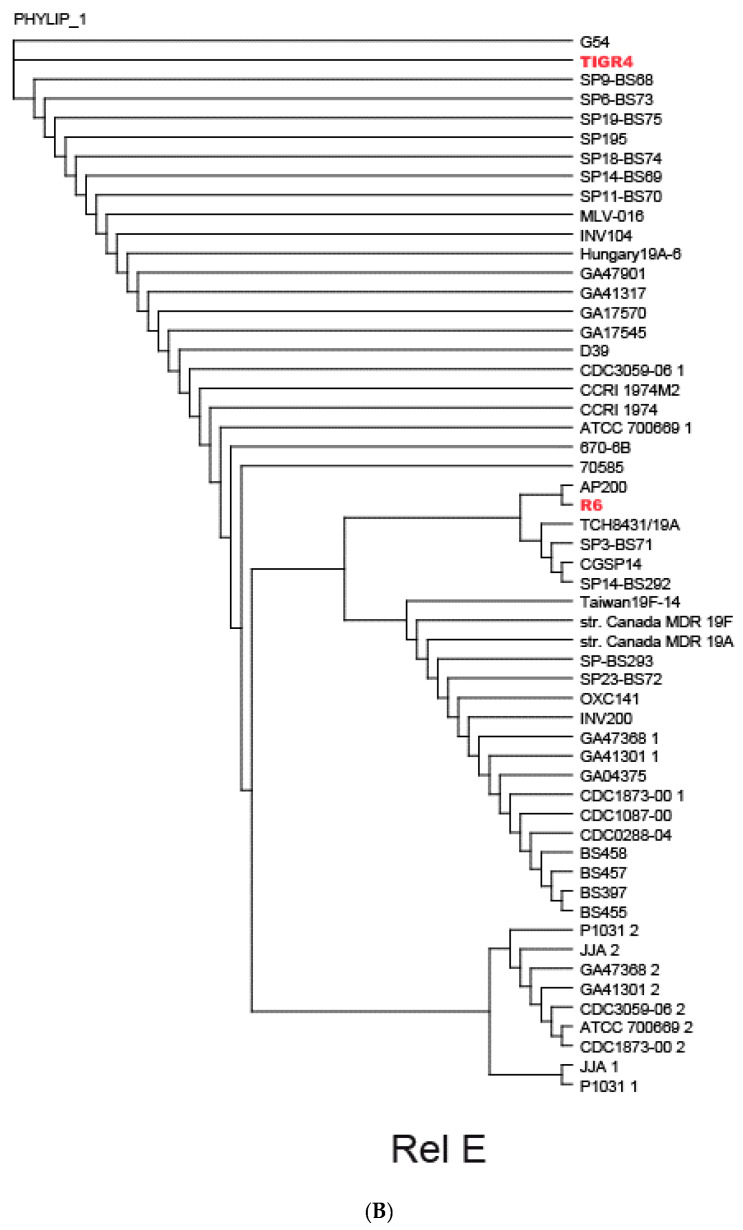
(**A**). Alignment of RelE proteins different pneumococcal strains. BLASTp using the pneumococcal RelE amino acid sequence from strain R6 as query retrieved 74 RelE alleles, of which we selected the 40 depicted ones as representatives of the best-characterized strains [41]. Three groups were found with 87, 84, and 87 residues, respectively. The first group (87 residues) varied significantly from the other two, with amino acid changes scattered around the protein sequence (see the conservation diagram, in yellow, below). The second sets of RelE proteins have 84 residues, in which strain TIGR4 is included (red letters). The last six strains (87 residues long) showed the RelE proteins having an MNN N-terminal extension, the group represented by strain R6 (red letters). However, these three N-terminal residues were present only in 5% of the purified pneumococcal protein, whereas the 95% rest lack these three residues, as determined by MALDI assays (Figure 1B and Supplementary Figure S1). Below the alignments, the conservation and quality (yellow), the consensus (black) and the occupancy (grey) are depicted in a diagram from the Jalview 2.0 program [51]. (**B**). Phylogenetic tree of the 40 selected pneumococcal toxins RelE generated by CLUSTAL Omega program (five iterations). The positions of the toxins in the two most representative pneumococcal strains, R6 and TIGR4, are indicated in boldface red letters.

**Figure 3:**
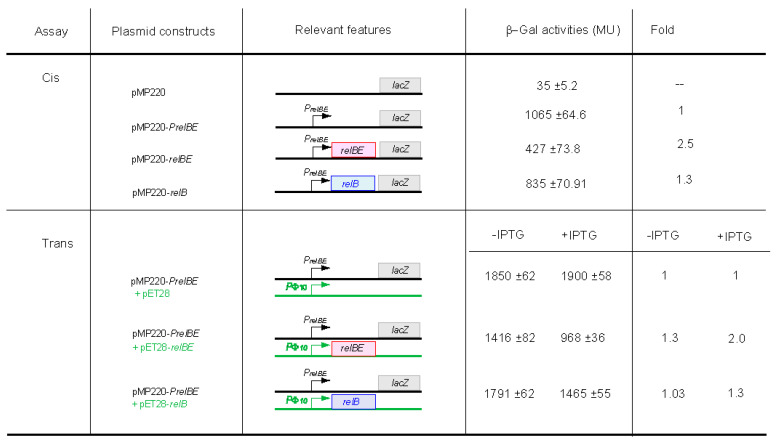
Control of expression by RelB and RelB:RelE proteins measured by β-galactosidase expression. Schematic diagrams of the recombinant plasmids carrying various *relBE*-*lacZ* transcriptional fusions, and their corresponding β-galactosidase activities. DNA fragments containing combinations of the *relBE* locus were cloned upstream of the promoterless *lacZ* gene (grey box) in pMP220, resulting in the various pMP220-derived recombinants as shown in the assays. When either the *relBE* or the *relB* products were supplied in trans, their synthesis was directed by the T7 Φ10 promoter, inducible by IPTG, under which the pneumococcal genes were cloned into the compatible plasmid pET28. Fold indicates repression-fold, being 1 the values obtained from cultures carrying both the pMP220-P*_relBE_* plasmid vector and the pET28 ‘empty’ vector.

*Escherichia coli* BL21(DE3) Codon-Plus RIL (F^−^, *lon*, *omp*T, *hsd*S{*r_B_^−^ m_B_^−^*} *dcm^+^*, Tet^r^, *gal l*, *end*A, Hte {*argU ileY leuW* Cam^r^}), Stratagene (San Diego, California, USA) was used for overexpression and purification of RelB:RelE protein complex and RelB protein alone. This strain has a single copy of the phage T7 RNA polymerase gene under the control of the IPTG-inducible *lacUV5* promoter [42]. Further, this strain has been engineered to express proteins with a high level of AGG/AGA (arginine), AUA (isoleucine), and CUA (leucine) codons, as is the case for the RelB and RelE proteins. The strain *E. coli* JM109(DE3) (λ DE3, *end*A1, *rec*A1, *gyr*A96, *thi, hsd*R17 (r_k_^−^, m_k_^+^), *rel*A1, *sup*E44, Δ(*lac-pro*AB), (F’, *tra*D36, *pro*AB+, *lac*I^q^ZΔM15); Promega, Madison, Wisconsin, USA) was employed for the β-galactosidase assays. The source of chromosomal DNA for amplification and cloning of the pneumococcal *relBE* operon was *S. pneumoniae* R6 (wild type, a non-capsulated derivative of strain D39 [43]) (Figure 1, Figure 2 and Figure 3).

*E. coli* cultures were grown at 37 °C in Tryptone-Yeast extract (TY) medium [44] supplemented with 30 µg/mL kanamycin (Km), 30 µg/mL chloramphenicol (Cm), or 2 µg/mL tetracycline (Tc). The strains were transformed by electroporation as described [45], using a Micro Pulser (BioRad, Madrid, Spain) at 25 μF, 2.5 kV/cm y 200 Ω. Pneumococcal cultures were grown at 37 °C on AGCH semi-synthetic medium, as reported in Reference [46].

### 2.2. Plasmid Constructions

The expression vector used for protein purification was plasmid pET28 (Novagen, Madrid, Spain, conferring resistance to Km, Km^R^). On this plasmid, either the entire *relBE* operon (pET28*relBE*, 5736 base pairs, bp) or the antitoxin gene (pET28*relB*, 5579 bp) were cloned and placed under the control of promoter Φ10 of phage T7. Due to its high toxicity, the gene encoding the pneumococcal toxin could not be cloned alone. Plasmid pET28*relBE* yielded an untagged antitoxin and a C-terminal His_6_-tagged toxin, whereas plasmid pET28*relB*, the antitoxin gene was the one with a His_6_-tag placed at its N-terminal end. The construction of these two plasmids has been reported previously [1].

To perform the transcriptional fusion assays, plasmid pNM220 conferring Tc^R^ [29] was used as the vector. The recombinant plasmids constructed carried different regions of *relBE* operon amplified by PCR from the chromosome of *S. pneumoniae* R6, and were transcriptionally coupled to the *lacZ* gene. Constructions were as follows:

- pMP220-P*_relBE_* (10,694 bp) carry a 210-bp PCR DNA fragment with the promoter region of the pneumococcal *relBE* operon. The primers used were: relBE-184E (5′-CGGGATCCGAACTGGCTCATATGACCATGG-3′) and relBE+28K (5′-CGGGGTACCCATCTTTTGTGTCCCTTTTTTAATG-3′).
- pMP220-*relBE* (11,190 bp) carry a 706-bp DNA fragment containing the promoter and the entire *relBE* operon. The primers used were: relBE-184E (5′-CGGGATCCGAACTGGCTCATATGACCATGG-3′) and relECK (5′-CGGGGTACCTCAATAAATATCTCTCCGATGACC-3′).
- pMP220-*relB* (10,934 bp) carry the promoter region and gene *relB* in a DNA fragment of 450 bp. Primers used were: relBE-184E (5′-CGGGATCCGAACTGGCTCATATGACCATGG-3′) and relB2CK (5′-CGGGGTACCTTATTCATCCTTCAAGCCTAAATC-3′).

### 2.3. β-Galactosidase Activity Measurements

To perform *cis-*transcriptional regulation experiments, *E. coli* JM109(DE3) cells were transformed with DNA from the plasmids carrying the pMP220 replicon, whereas *trans*-transcriptional regulation assays were done by using cells harbouring plasmid pMP220-*P_relBE_* that were transformed with DNA from the plasmid-derivatives with the pET28 replicon (see also the results in Figure 3). In all cases, cells harboring plasmids were grown aerobically at 37 °C to middle exponential phase (OD_600_ ~0.4) and, in the case of *trans*-complementation experiments, expression of the pneumococcal proteins was induced by addition or not of 1 mM IPTG, 30 min before the measurements. The β-galactosidase activities were determined as reported [47], but the cultures were dispensed in a 96-well microplate (EIA/RIA flat-bottom plates, Corning, New York, NY, USA) and absorbance data were collected with a Varioskan Flash reader (Thermo-Scientific, Madrid, Spain). The β-galactosidase-specific activities were calculated in Miller units (MU). Each experiment was repeated in at least three independent experiments. Statistics were calculated with the aid of the SigmaPlot program. 

### 2.4. Overexpression and Purification of Pneumococcal Proteins RelB-RelE and RelB

Expression and purification of RelB:RelEHis_6_ and His_6_RelB were performed, essentially as described in Reference [48]. Briefly, RelB and RelEHis_6_ were purified as a complex of both proteins from cells harbouring plasmid pET28*relBE*, whereas the antitoxin His_6_RelB was purified from cells harbouring plasmid pET28*relB*. Cells from 2 L cultures carrying either plasmid were grown to late exponential phase and induced by the addition of 0.7 mM IPTG (30 min, 30 °C), followed by the addition of rifampicin (200 µg/mL, 90 min). Cells were collected by centrifugation, washed twice and resuspended in 40 mL of buffer C (20 mM Tris pH 8.0, 5% ethylene glycol, 10 mM imidazole, 1 mM β-mercaptoethanol, 500 mM NaCl) to which two tablets of protease inhibitor cocktail (Roche) were added. The cell paste was passed twice through a French pressure cell, and the lysate was cleared by low-speed centrifugation to remove unbroken cells and cell debris (30 min, 6000× *g*, 4 °C). The supernatants were loaded onto a nickel column (His-select Nickel Affinity Gel, Sigma-Aldrich, Madrid, Spain) that was washed with buffer C, and the proteins were eluted in buffer E (20 mM Tris pH 8.0, 5% ethylene glycol, 250 mM imidazole, 1 mM β-mercaptoethanol, 500 mM NaCl). Fractions were analyzed by gel electrophoresis on 16% SDS-Tricine-polyacrylamide gels (SDS-PAGE). Proteins were detected by staining with Coomassie Brilliant Blue R-250 (Bio-Rad, Madrid, Spain). Fractions containing the peaks of the desired proteins were pooled, dialyzed against buffer S (20 mM Tris pH 7.6, 1 mM EDTA, 5% ethylene glycol, 1 mM DTT, 500 mM NaCl), and applied to a gel filtration column (Superdex 200 XK16/60, Amersham Biotech, Madrid, Spain), and the fractions containing the desired proteins were pooled and concentrated by filtration through 3 kDa cut-off filters (Pall, Port Washington, New York, NY, USA). The proteins were stored at −80 °C.

### 2.5. DNase I and Hydroxyl Radical Footprinting Experiments

A 199-bp fragment of the *S. pneumoniae* R6 chromosome was PCR-labelled at the 5′-end of one (coding) or the other (non-coding) strands, respectively, using primers labelled with (γ-^32^P)-ATP (3.000 Ci/mmol; PerkinElmer, Waltham, MA, USA) and T4 polynucleotide kinase (New England Biolabs, Ipswich, MA, USA). The ^32^P-labelled DNA fragment was incubated with increasing concentrations of either RelB:RelEHis_6_ protein complex or His_6_RelB protein in DNase I buffer (40 mM Tris-HCl, pH 7.9, 6 mM MgCl_2_, 1 mM CaCl_2_, 10 mM NaCl), supplemented with 1 mM DTT, 10% glycerol, 100 mM NaCl and 10 µg/mL heparin or 50 ng Poli(dI-dC), in a final volume of 50 µL. After 20 min at room temperature, 0.04 units of DNase I (Roche Applied Science, Sant Cugat del Vallès, Barcelona, Spain) were added and reactions continued for 5 min more. DNase I digestion was stopped by addition of 25 µL Stop solution (2 M ammonium acetate, 0.15 M EDTA, 0.8 mM sodium acetate pH 7, 400 µg/m tRNA). DNA was precipitated with ethanol, dried and dissolved in loading buffer (95% formamide, 20 mM EDTA, 0.05% bromophenol blue and 0.05% xylene cyanol). Samples were heated at 80 °C, 3 min and loaded onto 7 M urea-8% PAA gels and run together with the sequencing chemical reactions of the same fragment [49]. Gels were dried and detected using a Fujifilm Image Analyzer FLA-3000 or by autoradiography. The intensity of the bands was quantified using the ImageLab software, v. 5.2.1, BioRad Laboratories, Madrid, Spain).

Hydroxyl radical (OH·) footprinting experiments were performed using the same ^32^P labelled 199-bp DNA fragment, which was incubated with increasing concentrations of the proteins in 100 mM NaCl and 10 µg/mL heparin or 50 ng Poli(dI-dC), 20 min at room temperature. The OH· cleavage of DNA was initiated by the addition of 9 µL of the reactive mixture (Fe(II)-EDTA 10×, H_2_O_2_ and sodium ascorbate 10×), essentially as reported [50]. After 7 min at room temperature, the reactions were stopped by the addition of 14.7 µL of stop solution (0.041 M thiourea, 1.5 M NaAc pH6, 0.68 mg/mL tRNA). The DNA samples were ethanol-precipitated, dissolved in loading buffer and applied to a sequencing gel, as above.

### 2.6. Bioinformatics Analyses

The pneumococcal strains encoding the *relE* toxins were retrieved by PSI-BLAST using those encoded by strain R6 [1] as a query. Alignment of the pneumococcal RelE proteins was performed by the use of BLASTp (NCBI), followed by the selection of the proteins encoded by the best characterized pneumococcal strains ([41]; see the results in Figure 2). This subset of pneumococcal RelE proteins was aligned by the use of the CLUSTAL Omega program (https://www.ebi.ac.uk/Tools/msa/clustalo/; accessed on 2 April 2021), and the output was redrawn by the use of Jalview 2.0 program [51]. The CLUSTAL Omega program was also used to generate the phylogenetic trees of *relE* genes, using the default values and five iterations.

## 3. Results

### 3.1. The relBE Operon of Streptococcus pneumoniae

The pneumococcal *relBE* operon is a compact region that is present in, at least, one copy in all the pneumococcal genomes analysed (Figure 1). The polymorphisms found in the chromosomal regions around the *relBE* operon [1,36] did not seem to affect the activity of the operon in the sense that it was flanked by transcriptional terminators upstream and downstream of the genes [38]. In the case of strain R6, which is the one used here, is the operon locus is termed *spr1104* and *spr1103* (genes *relB* and *relE*, respectively; [26]) and it is placed between the *vicX* gene (encoding a metal-dependent hydrolase) and a type II restriction/modification methylase and endonuclease. Separating these latter two genes from the *relBE* operon, a 107-nucleotide long RUP of unknown function [52,53] was always observed [1]. 

The *relBE* operon is transcribed from a single promoter, P*_relBE_* [29] and two palindromic sequences (PS1 and PS2, Figure 1A) encompass its −10 region. The ribosome-binding site (RBS) of *relB* is nearly canonical for *S. pneumoniae,* as derived from 87 examples of RBSs reported for strain R6 (http://www.changbioscience.com/primo/ti.html, accessed on 29 January 2021), whereas a short RBS for *relE* (5′-GGAGGA-3′) is embedded within the last codons of *relB* (Figure 1A). Further, the intergenic space between *relB* and *relE* is such that the stop codon of *relB* is placed after the *relE* open reading frame, a situation indicative of translational coupling. The RelB protein (80 residues, pI = 4.37) and the RelB:RelE protein complexes encoded by strain R6 were purified, N-terminally sequenced, and their features were determined by analytical ultracentrifugation and native mass-spectrometry [48] and, later on, by MALDI-TOF experiments [54]. We found that most (~90%) of RelB lacked the first Met residue, whereas most (~95%) RelE toxin (87 residues, pI = 10.27) lacked the first four amino acids (MNNL) due to an internal initiation of translation codon (Figure 1B and Supplementary Figure S1). Due to its high toxicity, we could not clone the *relE* gene alone, even in the presence of high doses of the antitoxin provided *in trans* [48].

### 3.2. Distribution of the Pneumococcal Toxin RelE

BLASTp searches with the RelE (formerly RelE2; [29,38]) protein of strain R6 as the query, retrieved 74 hits from the pneumococcal genomes, as well as many more from other *Streptococcus* species, such as *S. mitis*, *S. oralis*, *S. suis*, *S. pseudopneumoniae,* and *S. tigurinus*. We selected 40 of the RelE proteins because they are encoded by the most representative strains of pneumococci [41]. They were aligned using CLUSTAL Omega and the results were drawn by Jalview 2.0 programs (Figure 2A). The results showed that the pneumococcal RelE proteins were associated with three different groups, the first two being highly similar except for the first four residues that were present in the group represented by strain R6 and missing in the most populated group represented by strain TIGR4 (Figure 2A). Thus, we propose that only two main families of RelE proteins are encoded by the pneumococcal chromosome, the first one having 84 residues, and the second one 87 residues with several amino acid changes scattered along all the protein sequence (Figure 2A). Using the same CLUSTAL Omega program, we retrieved a phylogenetic tree of the RelE proteins that showed that they are present in the most representative pneumococcal strains, R6 and TIGR4, although they were phylogenetically separated (Figure 2B). Nevertheless, if we consider that only 5% of the purified proteins exhibited the N-terminal MNN-extension (Figure 1B and Supplementary Figure S1), this supposed separation could be neglected. 

### 3.3. The Pneumococcal RelB Protein Represses Transcription of the relBE Operon and RelE Acts as an Efficient Co-Repressor

The initiation of transcription of the pneumococcal *relBE* operon was determined and only one transcript was found, indicative of the presence of a single promoter [29]. However, whether this promoter was subjected to regulation by RelB antitoxin or RelB:RelE pair, as in another type II TAs [25,30], was unknown. To approach this question, transcriptional fusions were constructed, having a promoterless *E. coli lacZ* gene as a reporter. To discard any ambiguity, we followed two approaches: (i) *cis* assays in which the P*_relBE_* promoter was directing the synthesis of *relB* or *relBE* transcriptionally coupled to *lacZ*, and (ii) *trans*-complementation assays in which promoter P*_relBE_* was fused to *lacZ,* but uncoupled from its genes that, in turn, were placed under the control of the IPTG-inducible promoter Φ10 from phage T7, which was provided by another compatible plasmid. The results and schematic representations of the various constructions are depicted in Figure 3. It was apparent that RelB acted as a weak repressor in both tested conditions. The differences between repressed versus unrepressed conditions were small (around 1.3 fold), but the values were statistically significant (*p* < 0.05). The incorporation of RelE to the transcriptional fusions, i.e., the RelB:RelE protein complex, led to a twofold increase in the repression levels, demonstrating that RelE toxin acted as a co-repressor to further increase transcriptional regulation from the P*_relBE_* promoter. Such a transcriptional regulation was not observed for the pneumococcal *yefM-yoeB* operon (also belonging to the RelBE family of pneumococcal TAs; [34,35]). In this case, the synthesis of YefM and YoeB proteins was directed by two different promoters, one of them regulated by the combined action of YefB:YoeB protein complex, whereas the second, weak promoter was unregulated and contributed to keeping a basal level of TAs [30].

### 3.4. Determination of DNA Binding Sites of RelB Protein and the RelB:RelE Protein Complex 

**Figure 4:**
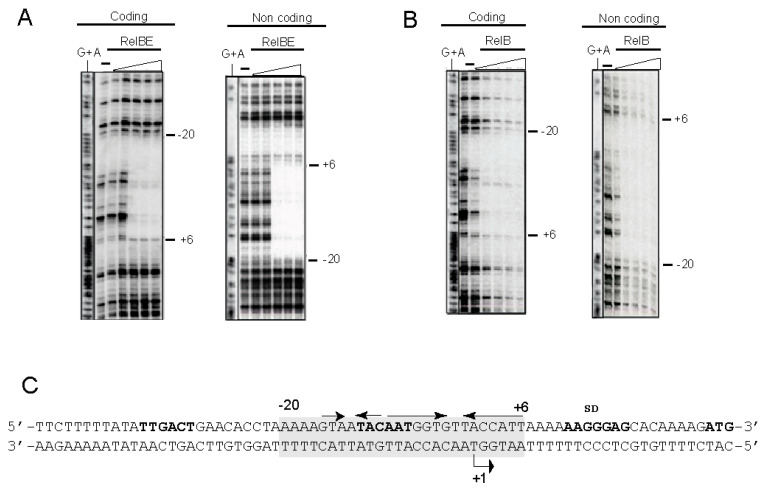
Binding of RelB and RelB:RelE to their DNA target assessed by footprinting with DNase I. DNase I footprints generated by RelB:RelE complex (**A**) and RelB protein (**B**) on the coding and non-coding strands of the labelled DNA fragment encompassing the P*_relBE_* promoter (**C**, boldface letters). Concentrations of RelB used were of 5, 10, 15, 20, and 30 μM, whereas those of the RelB:relE complex were of 0.4, 0.8, 1.0, 2.0, and 5 μM. Reaction samples were run on 8% polyacrylamide gels, where (−) indicate the controls completed in the absence of the proteins; G+A are the sequencing reactions performed on the same DNA fragment labelled at the coding and non-coding strands. (**C**) Shows a schematic representation of the DNase I footprints (shadowed, from positions −20 to +6 respective of the transcription start point, +1) on the promoter DNA sequence relative to the position of the two palindromic sequences (arrows with arrowheads pointing to the symmetry axis). −35 and −10 boxes, transcription initiation point (+1), SD sequence and initiation codon of RelB are shown in boldface letters.

Previous analyses performed by electrophoretic mobility shift assays, native mass spectrometry, and analytical ultracentrifugation showed that the pneumococcal TA complex was bound to DNA at a region that encompasses the promoter of the *relBE* operon in a configuration compatible with a heterohexamer composed of two RelE and four RelB protomers [48]. The arrangement of the DNA region surrounding the *relBE* promoter showed the presence of two palindromic sequences, PS1 and PS2, of 6 bp and 3 bp, respectively, that overlapped the −10 sequence of the promoter and the transcription start site (see Figure 1). Similar short palindromes have also been identified in the promoter regions of other TAs [55] and identified as the binding sites of the proteins. These palindromes seem to play a role in ensuring efficient transcriptional repression of the TA operon [6,13,56]. To define precisely the positioning of the pneumococcal proteins on the DNA promoter region, we performed DNase I footprinting assays. A 199 bp ^32^P-labelled DNA fragment (including the *relBE* control sequences) was incubated with RelB:RelE and with RelB purified proteins; the controls did not receive any protein. The assays were performed on both, the coding and the non-coding strands (Figure 4). The results showed that RelB in complex with RelE (Figure 4A) or RelB alone (Figure 4B) generated a long footprint that extended from position −20 to +6 relative to the transcription initiation point (Figure 4C) in the coding strand; the same footprint was mirrored in the non-coding strand. Thus, the footprints generated by the proteins spanned the two inverted repeats, the −10 region of the P*_relBE_* promoter, and the transcription initiation site (Figure 4C). However, the footprint generated by protein RelB alone was less marked in both strands, and nearly 10 times more proteins to observe a footprint (10 µM) were needed as marked as the footprint generated by the RelB:RelE complex (1 µM).

### 3.5. High-Resolution Footprinting Assays 

**Figure 5:**
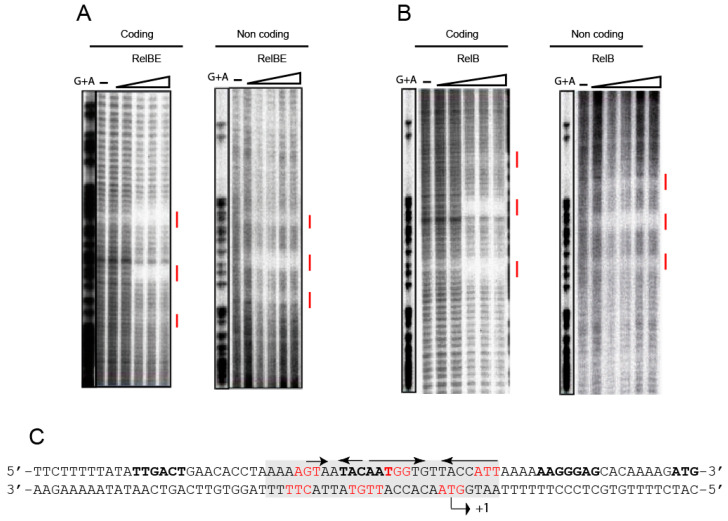
High-resolution footprints of RelB:RelE complex and RelB alone on their DNA target. The footprints (vertical red lines) were generated by incubation of the DNA-protein samples followed by breakage of the sugar-DNA backbone by the generation of hydroxyl radicals. RelB:RelE complex (**A**) and RelB protein (**B**) on the coding and non-coding strands of the labelled DNA fragment encompassing the P*_relBE_* promoter (**C**, boldface letters). The amounts of proteins used were the same as in Figure 4. Reaction samples were run on 8% polyacrylamide gels, where (−) indicate the controls done in the absence of the proteins; G+A are the sequencing reactions performed on the same DNA fragment labelled at the coding and non-coding strands. (**C**) Shows a schematic representation of the OH· footprints (red letters) on the promoter DNA sequence relative to the position of the two palindromic sequences (arrows with arrowheads pointing to the symmetry axis). −35 and −10 boxes, transcription initiation point (+1), SD sequence and initiation codon of RelB are shown in boldface letters.

The large footprint that the proteins have on their target DNA, as determined by the above DNase I experiments, provide information of the region where the proteins positioned on the DNA. However, since DNase I is a bulky enzyme, these footprints do not provide detailed information on the bases contacted by the proteins. To obtain an accurate knowledge of the DNA binding sites occupied by the proteins, a high-resolution footprint must be obtained. This can be achieved by small molecules like those generated by chemical attack on the sugar-phosphate backbone of the target DNA by free OH· radicals [57]. This technique provides minute information on the DNA binding sites of the desired proteins. A high-resolution profile of the binding sites of the proteins on the DNA target was thus achieved by the use of OH·-cleavage of DNA-RelB:RelE complexes [30,50,57]. The same ^32^P-labelled DNA fragment used above was bound to either the RelB:RelE protein complex or to RelB alone, followed by the generation of OH· radicals [57]. The results (Figure 5) showed that all the footprints generated by the DNase I could also be detected by OH·-chemical cleavage. However, the long footprints observed in both strands (Figure 4A,B) were divided into smaller footprints (Figure 5A,B). No further regions protected by the proteins could be observed even in gels run for longer periods. Three distinct protections on the coding strand were observed with RelB alone or RelB:RelE complex and each of the three covered three bases. On the non-coding strand, the protection pattern was of three, four, and three bases protected. The footprints detected were the same independently of the proteins used, whereas those by RelB were weaker than those generated by the RelB:RelE complex, as also observed with the DNase I experiments (Figure 4C and Figure 5C). 

## 4. Discussion

The pneumococcal *relBE* operon is present in all the strains sequenced so far, indicative of the relevance of this TA pair in the bacterial lifestyle and despite the variability of the genetic context of the operon in the different strains analyzed [38]. While the toxin counterpart is the most conserved protein of the TA pair, some variability was observed in the amino acid sequence of the RelE toxin, especially in the N-terminal and in the central regions of the protein where insertion of three residues, MNN and TTN, respectively, was observed (Figure 2). Whether these changes reflect a difference in the activity of the toxin is, at present, unknown. It has been proposed that the functionality of the bacterial TA systems might be linked to them being horizontally transferred [14]. This could appear to be the case in some of the pneumococcal RelBE operons present in several clinical isolates because of the presence of *IS*s genetic elements or rearrangements around the region [1,36]. There is a lack of information on how these TAs are transferred. A hint could be derived from the finding that deletion of the PezAT system (present in the pneumococcal Pathogenicity Island 1) led to enhanced genetic competence, among other features [28]. 

**Figure 6:**
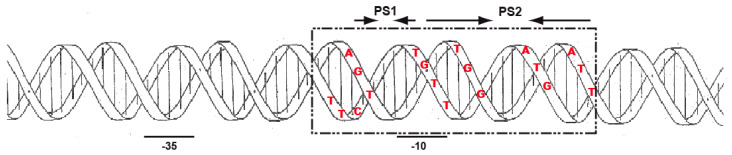
Summary of the footprints generated by the RelB:RelE protein complex on the DNA surrounding the P*_relBE_* promoter region. The DNA region is shown as having 10.5 bp per helical turn [58,59]. The dashed square represents the DNase I footprints, whereas the red letters are the bases whose deoxyriboses are protected by the protein from the OH· radicals. The relative positions of the −35 and −10 regions are indicated by a horizontal solid line, whereas the two palindromic sequences, PS1 and PS2, are indicated by arrows with the arrowheads pointing to the symmetry center.

From the experiments presented here, we can conclude that in vivo and in vitro, that the binding of antitoxin RelB protein to DNA is weaker than that of the complex RelB:RelE, supporting a co-repressor role of RelE on the control of the operon expression reported earlier [48] and in agreement with reports from several type II TAs (reviewed by [13]). The high-resolution footprinting assays provided further information on the position of the proteins on the operator region: the central base of each footprint was separated by 10–11 bases to the next one, and they were displaced 1 base between coding and non-coding strands (summarized in Figure 6). This is the signature of proteins that bind to the DNA across the minor groove through the same face of the DNA helix [57], and RelB:RelE was bound on 2.5 consecutive helix turns, assuming a helical periodicity of 10.5 bp per helical turn [58,59]. Previous results from our laboratory [48] using analytical ultracentrifugation and native mass spectroscopy showed that antitoxin RelB existed mostly as a dimer, although a minority of monomers were also detected. When the RelB:RelE complex was analysed, we found that a majority of the complex generated mostly a heterohexamer {(RelB)_2_-(RelE)_2_-(RelB)_2_}, although minor species of heterotrimers {(RelB)_2_-(RelE)_1_} and heterododecamers (a dimer of heterohexamers) were also found. These results, in conjunction with the footprints presented here, show that the pneumococcal RelB:RelE complex binds to DNA as a heterohexamer. These observations are further supported by the three-dimensional structure of the RelBE complex of *E. coli*, in which two heterotrimers of (RelB)_2_:RelE is the more congruent structure of the proteins associated with the operator sequence, explaining, at the structural level, the basis of conditional cooperativity [60]. The complex showed a V-shaped structure that agrees with those found for other solved RelB:RelE complexes [60,61]. Whether this shape facilitates the DNA binding of the protein complexes to their target DNA and whether the proteins cause a DNA bend upon binding is still unknown. Interestingly, the model derived from the *E. coli* RelB:RelE structure agrees with the existence of a secondary, weak, DNA binding site for the protein complex [60], an observation derived from electrophoresis mobility shift assays [62]. In the case of the pneumococcal RelBE proteins, DNA band-shift assays [48], and the present DNA footprints do not support the existence of a secondary DNA binding site in the operator region to control the expression of the *relBE* proteins, because the increase in the amount of proteins used did not show any further protected region (Figure 4 and Figure 5). An alternative possibility stems from our results with native-mass spectrometry assays, which showed that the heterohexamer harbored two protomers, each of them composed of one RelB dimer and one RelE monomer [48]. Changes in the ratio of the antitoxin and toxin molecules would lead to various degrees of protein oligomerization, pointing to a very agile regulation of the operon and so providing a fast response to changes in the bacterial environment, as shown for the *relBE* homologs *yefM-yoeB* and *axe-txe* TAs modules [63]. 

The solution of the structure of the pneumococcal RelB and RelB:RelE proteins alone and in complex with their target DNA could provide better insights into the interaction of these proteins with their target DNA, as has been the case for the *Staphylococcus aureus yefM-yoeB* TAs [64]. 

## Data Availability

Not applicable.

## Supplementary Materials

The following are available online at https://www.mdpi.com/article/10.3390/microorganisms9040851/s1, Figure S1: MALDI-TOF spectra of the pneumococcal RelB and RelE proteins with the two peaks obtained for each one of the proteins (molecular mass indicated on top of the peaks). RelB purified mainly as lacking the first Met residue, probably processed by the E. coli strain used for its overexpression. RelE lacked the first three amino acid residues (Met-Asp-Asp) most likely as the result of internal initiation at the fourth codon (TTG, translated as Met). Below the nucleotide sequence at the initiation of translation region of toxin RelE is shown with the ATG initiation codon (red) predicted to be the first one translated and the determined N-terminal sequence of RelE.
