# Supplementary material for: Interactions of the Streptococcus pneumoniae Toxin-Antitoxin RelBE Proteins with Their Target DNA
Source: Microorganisms. 2021 Apr 15;9(4):851. doi: 10.3390/microorganisms9040851 (PMC8071376; doi:10.3390/microorganisms9040851)
Supplement: Supplementary file 1 [file microorganisms-09-00851-s001.zip › microorganisms-1160345-supplementary.pdf]

SUPPLEMENTARY MATERIAL

Interactions of the *Streptococcus pneumoniae* Toxin-Antitoxin RelBE Proteins with their Target DNA

Inmaculada Moreno-Córdoba, Wai Ting Chan, Concha Nieto, and Manuel Espinosa

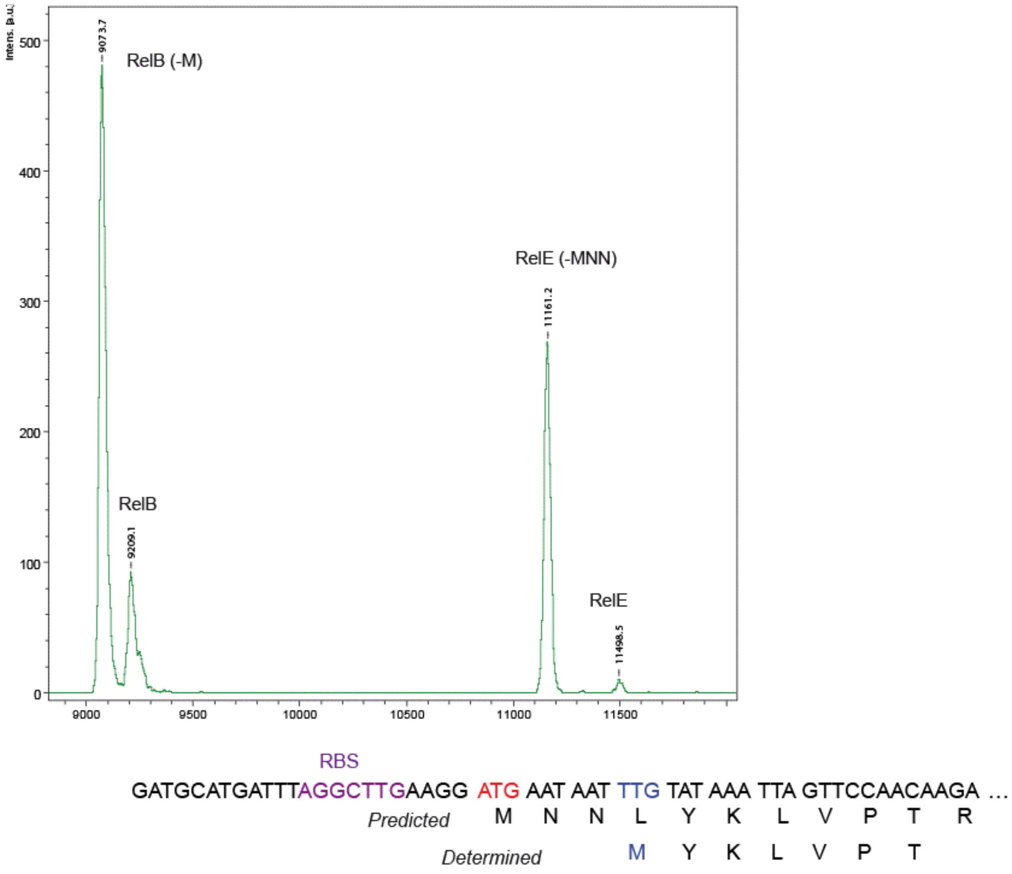

Supplementary Figure S1. MALDI-TOF spectra of the pneumococcal RelB and RelE proteins with the two peaks obtained for each one of the proteins (molecular mass indicated on top of the peaks). RelB purified mainly as lacking the first Met residue, probably processed by the *E. coli* strain used for its overexpression. RelE lacked the first three amino acid residues (Met-Asp-Asp) most likely as the result of internal initiation at the fourth codon (TTG, translated as Met). Below the nucleotide sequence at the initiation of translation region of toxin RelE is shown with the ATG initiation codon (red) predicted to be the first one translated and the determined N-terminal sequence of RelE.
